# Fasting Enhances the Contrast of Bone Metastatic Lesions in ^18^F-Fluciclovine-PET: Preclinical Study Using a Rat Model of Mixed Osteolytic/Osteoblastic Bone Metastases

**DOI:** 10.3390/ijms18050934

**Published:** 2017-04-29

**Authors:** Shuntaro Oka, Masaru Kanagawa, Yoshihiro Doi, David M. Schuster, Mark M. Goodman, Hirokatsu Yoshimura

**Affiliations:** 1 Research Center, Nihon Medi-Physics Co., Ltd., 3-1 Kitasode, Sodegaura, Chiba 299-0266, Japan; masaru_kanagawa@nmp.co.jp (M.K.); yoshihiro_doi@nmp.co.jp (Y.D.); hirokatsu_yoshimura@nmp.co.jp (H.Y.); 2 Division of Nuclear Medicine and Molecular Imaging, Department of Radiology and Imaging Sciences, Emory University, Atlanta, GA 30329, USA; dschust@emory.edu (D.M.S.); mgoodma@emory.edu (M.M.G.)

**Keywords:** bone metastasis, breast cancer, FACBC, fluciclovine, FDG, positron emission tomography, prostate cancer

## Abstract

^18^F-fluciclovine (*trans*-1-amino-3-^18^F-fluorocyclobutanecarboxylic acid) is an amino acid positron emission tomography (PET) tracer used for cancer staging (e.g., prostate and breast). Patients scheduled to undergo amino acid-PET are usually required to fast before PET tracer administration. However, there have been no reports addressing whether fasting improves fluciclovine-PET imaging. In this study, the authors investigated the influence of fasting on fluciclovine-PET using triple-tracer autoradiography with ^14^C-fluciclovine, [5,6-^3^H]-2-fluoro-2-deoxy-d-glucose (^3^H-FDG), and ^99m^Tc-hydroxymethylene diphosphonate (^99m^Tc-HMDP) in a rat breast cancer model of mixed osteolytic/osteoblastic bone metastases in which the animals fasted overnight. Lesion accumulation of each tracer was evaluated using the target-to-background (muscle) ratio. The mean ratios of ^14^C-fluciclovine in osteolytic lesions were 4.6 ± 0.8 and 2.8 ± 0.6, respectively, with and without fasting, while those for ^3^H-FDG were 6.9 ± 2.5 and 5.1 ± 2.0, respectively. In the peri-tumor bone formation regions (osteoblastic), where ^99m^Tc-HMDP accumulated, the ratios of ^14^C-fluciclovine were 4.3 ± 1.4 and 2.4 ± 0.7, respectively, and those of ^3^H-FDG were 6.2 ± 3.8 and 3.3 ± 2.2, respectively, with and without fasting. These results suggest that fasting before ^18^F-fluciclovine-PET improves the contrast between osteolytic and osteoblastic bone metastatic lesions and background, as well as ^18^F-FDG-PET.

## 1. Introduction

The most used positron emission tomography (PET) tracer for cancer imaging is 2-deoxy-2-^18^F-fluoro-d-glucose (^18^F-FDG), which is an analogue of D-glucose. Cancer cells take up this PET tracer because ^18^F-FDG shares its transport routes (i.e., glucose transporters) with D-glucose. High concentrations of plasma glucose may compete with ^18^F-FDG at glucose transporters in cancer cells and elevates plasma insulin which unfavorably alters the biodistribution to the radiotracer [1]. Thus, patients scheduled to undergo ^18^F-FDG-PET are required to fast for 4 h to 6 h to lower plasma glucose concentration before PET imaging.

Several amino acid (AA) PET tracers, including ^11^C-methionine, have been used for diagnostic purposes in cancer patients [2]. Although studied previously, issues regarding the necessity of fasting in clinical PET with AA tracers [3] have yet to be resolved. Fasting is routinely recommended for patients undergoing AA-PET as well as ^18^F-FDG-PET. There have been few studies investigating the influence of fasting or food intake before AA-PET. The authors of one study investigating ^11^C-methionine-PET for head and neck cancer concluded that food ingestion may decrease ^11^C-methionine uptake in tumors, although the image quality remained satisfactory after food ingestion [4].

^18^F-fluciclovine, also known as *trans*-1-amino-3-^18^F-fluorocyclobutanecarboxylic acid (FACBC; code name: NMK36, Nihon Medi-Physics; brand name: Axumin, Blue Earth Diagnostics, Burlington, MA, USA), is an AA-PET tracer, and has been approved by the United States Food and Drug Administration for the detection of prostate cancer (PCa) recurrence since 2016. Even in ^18^F-fluciclovine-PET imaging, patients usually fast for ≥4 h before tracer administration without any conclusive evidence supporting the influence of fasting for ^18^F-fluciclovine uptake in tumors [5]. Although one clinical research study involving non-fasted breast cancer (BCa) patients has been published, PET imaging was performed for the chest only and no comparison was made to control fasted subjects [6]. Thus, it is not known whether fasting influences tumor uptake of ^18^F-fluciclovine in PCa and BCa patients.

Most patients with advanced PCa and BCa present with mixed osteolytic and osteoblastic bone metastases [7]. To investigate the influence of fasting on ^18^F-fluciclovine accumulation in bone metastatic lesions, we established a rat bone metastatic model, which forms both osteolytic and osteoblastic lesions in the tibia and/or femur, by intra-arterial injection of MRMT-1 cells, a rat BCa cell line with a 100% success rate for skeletal metastases. Using this animal model, triple-tracer autoradiography was performed using *trans*-1-amino-3-fluoro[1-^14^C]cyclobutanecarboxylic acid (^14^C-fluciclovine), [5,6-^3^H]-2-Fluoro-2-deoxy-d-glucose (^3^H-FDG), and ^99m^Tc-hydroxymethylene diphosphonate (^99m^Tc-HMDP) to compare the tracers’ accumulation at identical bony lesions. Our findings suggest that overnight fasting influenced the uptake of ^18^F-fluciclovine in the bony lesions, and that the tumor-to-muscle uptake ratios in osteolytic and osteoblastic lesions were higher in the fasted condition compared with the fed condition.

## 2. Results

### 2.1. In Vitro Experiments

**Figure 1:**
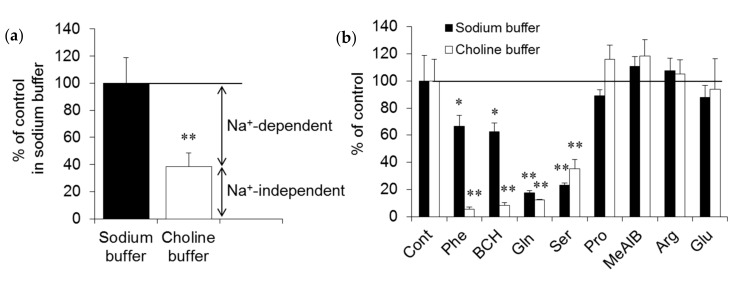
Characteristics of ^14^C-fluciclovine transport in MRMT-1 cells. (**a**) Contributions of Na^+^-dependent and -independent carriers on the uptake of ^14^C-fluciclovine. The transport of ^14^C-fluciclovine in sodium buffer was normalized to 100%. (**b**) Competitive inhibition of 10 μmol/L ^14^C-fluciclovine transport by naturally-occurring and synthetic amino acids (2.0 mmol/L). The control transport (absence of inhibitors) of ^14^C-fluciclovine in sodium and choline buffer was normalized to 100%. Each bar represents the mean ± standard deviation (SD) (*n* = 6). * *p* < 0.05, ** *p* < 0.01. Arg: arginine, BCH: 2-amino-bicyclo[2,2,1]heptane-2-carboxylic acid, Cont: control, Gln: glutamine, Glu: glutamate, MeAIB: 2-(methylamino)-isobutyric acid, Phe: phenylalanine, Pro: proline, Ser: serine.

^14^C-fluciclovine is a synthetic AA and is transported by AA transporters (AATs). To investigate which transport system mediates the transport of this tracer in MRMT-1 cells, in vitro uptake inhibition experiments were performed. As shown in Figure 1a, the uptake of ^14^C-fluciclovine decreased to 38.4% in choline buffer compared with sodium buffer, corresponding to contributions of the Na^+^-dependent and -independent carriers for ^14^C-fluciclovine transport in MRMT-1 cells of 61.6% and 38.4%, respectively. To narrow the subtypes of AATs involved in ^14^C-fluciclovine transport in MRMT-1 cells, competitive uptake experiments were performed in the absence or presence of several synthetic and naturally-occurring AAs as inhibitors. As shown in Figure 1b, small neutral AAs, such as glutamine and serine, showed strong inhibitory effects for ^14^C-fluciclovine uptake (82.4% and 77.0% decreases vs. control, respectively), while phenylalanine, a branched-chain AA, and 2-amino-bicyclo[2,2,1]heptane-2-carboxylic acid (BCH) demonstrated smaller inhibitory effects (33.3% and 37.6% decreases, respectively, vs. control) in the presence of sodium ion. In contrast, phenylalanine and BCH inhibited ^14^C-fluciclovine transport by more than 90% versus control in the absence of sodium ion. Proline, 2-(methylamino)-isobutyric acid (MeAIB), arginine, and glutamate had no statistically-significant inhibitory effect on ^14^C-fluciclovine transport into MRMT-1 cells. These results suggest that the strong inhibitory effects of glutamine and serine in the presence of sodium ion mean that the alanine-serine-cysteine (ASC) system, especially the ASC transporter 2 (ASCT2), is the primary carrier in Na^+^-dependent AATs. On the other hand, the intense inhibitory effects of phenylalanine and BCH in the absence of sodium ion suggest that system L, especially the L-type AA transporter (LAT1), is the primary carrier in Na^+^-independent AATs for the transport of ^14^C-fluciclovine in MRMT-1 cells.

### 2.2. In Vivo Experiments

**Figure 2:**
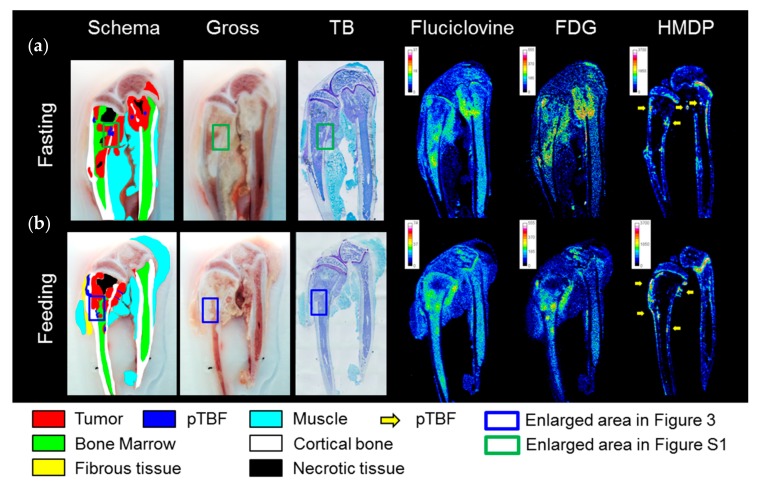
Triple-tracer autoradiography using ^14^C-fluciclovine, 2-deoxy-2-^18^F-fluoro-d-glucose (^3^H-FDG), and ^99m^Tc-hydroxymethylene diphosphonate (^99m^Tc-HMDP) in breast cancer bone metastasis model rats. Macroscopic images (schema, gross, toluidine blue (TB) staining) and autoradiograms of each tracer in (**a**) fasting and (**b**) fed rats are shown. Each image was adjusted for optimal contrast and color scale bars on each autoradiogram represent Bq range for each tracer. The high-power microscopic fields of typical osteolytic and osteoblastic lesions corresponding to the blue and green frames on the macroscopic images in Figure 2a,b are shown in Figure 3 and supplementary Figure S1, respectively. The lesions correspond to ^99m^Tc-HMDP-positive areas, except for physiological accumulation in growth plates, which were considered peri-tumor bone formation (pTBF) in osteoblastic lesions (yellow arrows).

The distribution patterns of ^14^C-fluciclovine in the tibia and/or femur were basically similar to ^3^H-FDG in both fasted and fed conditions, as shown in Figure 2. Visualization of the tumor mass in bone marrow cavities was clearer with ^3^H-FDG because the physiological accumulation of ^14^C-fluciclovine in bone marrow was higher than ^3^H-FDG. Although ^14^C-fluciclovine and ^3^H-FDG accumulated at the peri-tumor bone formation (pTBF) in osteoblastic lesions, characterized by ^99m^Tc-HMDP accumulation, the images were obscure compared with the tumor parenchyma (Figure 2).

**Figure 3:**
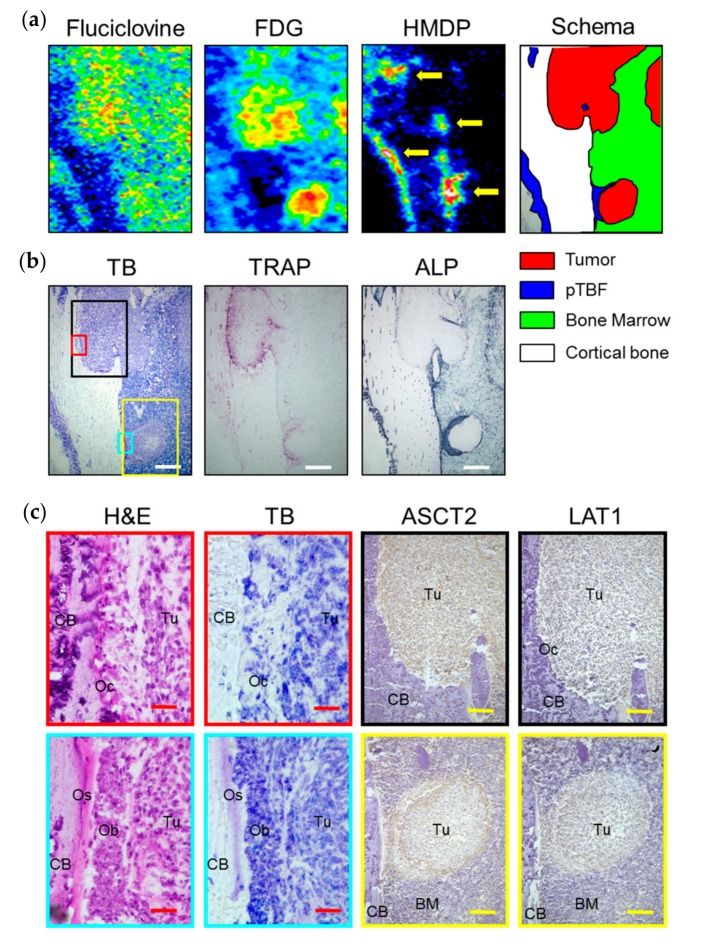
Comparison of the tracer accumulations of ^14^C-fluciclovine, 2-deoxy-2-^18^F-fluoro-d-glucose (^3^H-FDG) and ^99m^Tc-hydroxymethylene diphosphonate (^99m^Tc-HMDP), and the histological characteristics of typical osteolytic and osteoblastic lesions in a representative breast cancer bone metastasis model rat that was fed. (**a**) The enlarged autoradiograms and the schema and (**b**) the histological images (toluidine blue (TB), tartrate-resistant acid phosphatase (TRAP), alkaline phosphatase (ALP)) correspond to the blue frame on the schema in Figure 2b are represented. The lesions corresponding to ^99m^Tc-HMDP-positve were considered peri-tumor bone formation (pTBF) in osteoblastic lesions (yellow arrows). (**c**) The high-power microscopic fields (hematoxylin and eosin (H&E), TB, alanine-serine-cysteine transporter 2 (ASCT2), L-type amino acid transporter 1 (LAT1)) correspond to the black, red, yellow, and cyan frames on the TB image in Figure 3b are shown. The red, yellow, and white scale bars on each panel correspond to 50 μm, 200 μm, and 500 μm, respectively. BM: bone marrow, CB: cortical bone, Ob: osteoblasts, Oc: osteoclasts, Os: osteoids, Tu: tumor.

Figure 3 shows the results of histological examination in osteolytic and osteoblastic bone metastasis lesions in a representative rat that was fed. In the osteolytic lesion (black frame on the toluidine blue (TB) image in Figure 3b), the absorption pits with tartrate-resistant acid phosphatase (TRAP) activity indicating osteoclast infiltration was observed. Hematoxylin and eosin (H&E) staining revealed many osteoclasts, characterized by multiple nuclei on the surface of cortical bone in the pits (Figure 3c). On the other hand, the osteoblastic lesions (the yellow frame on the TB image in Figure 3b) revealed alkaline phosphatase (ALP) activity and pTBF, indicating the appearance of osteoblasts and osteoids, respectively, between tumor mass and the surface of cortical bone (H&E and TB images in Figure 3c). Almost all of the tumor cells in the osteolytic lesion were positive for ASCT2 (upper row in Figure 3c). On the other hand, ASCT2 expression in intra-tumoral cells was scant compared with that in the peripheral cells of the tumor mass and osteoblasts on the bone surface in the osteoblastic lesion (lower row in Figure 3c). LAT1-positive cells were scattered in all of the tumor tissues from both osteolytic and osteoblastic lesions (Figure 3c). No obvious differences were observed in the expression of ASCT2 and LAT1 between the fasted and fed rats (supplementary Figure S1). These results demonstrate that ^14^C-fluciclovine and ^3^H-FDG accumulated in histologically confirmed osteolytic and osteoblastic bone metastasis lesions.

**Figure 4:**
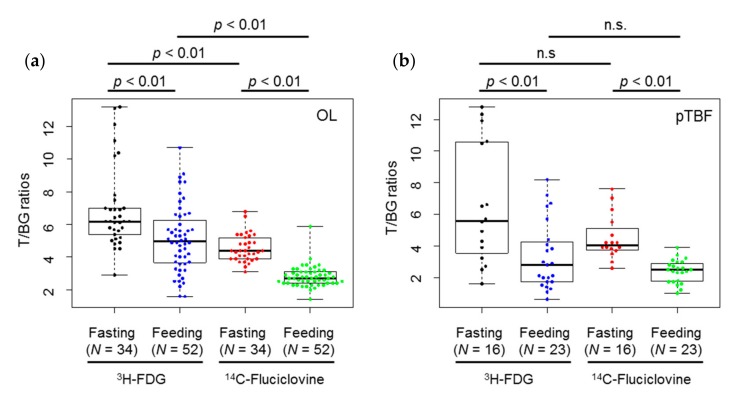
Beeswarm and box plots showing the distributions of target_mean_ (metastatic lesion)-to-background_mean_ (muscle) (T/BG) ratios of 2-deoxy-2-^18^F-fluoro-d-glucose (^3^H-FDG) and ^14^C-fluciclovine in (**a**) osteolytic lesions (OL), and (**b**) peri-tumor bone formation (pTBF) in osteoblastic lesions in breast cancer bone metastasis model rats, with and without overnight fasting. The numbers under each box indicate the number of lesions. n.s.: not significant.

Semi-quantitative analyses were used to evaluate tumor-to-muscle accumulation ratios of ^14^C-fluciclovine and ^3^H-FDG are shown in Figure 4 and Table 1. The target_mean_ (metastatic lesion)-to-background_mean_ (muscle) (T/BG) ratio of both tracers were statistically higher in the fasting condition than in the fed condition in osteolytic and pTBF lesions (*p* < 0.01). Comparing tracers, the ratios of ^3^H-FDG were statistically higher than ^14^C-fluciclovine in osteolytic lesions (*p* < 0.01), but not in pTBF lesions, under fasting and fed conditions. The distributions of T/BG ratios of ^3^H-FDG in both lesions were wider than that of ^14^C-fluciclovine, as shown in Figure 4 and Table 1.

## 3. Discussion

We aimed to determine whether fasting before ^18^F-fluciclovine-PET imaging improved visualization of bone metastasis lesions using a rat BCa bone metastasis model exhibiting osteolytic and osteoblastic lesions. Our findings suggest that fasting before fluciclovine-PET imaging improves the background contrast between osteolytic/osteoblastic bony lesions and muscle.

First, we investigated the AATs involved in the uptake of ^14^C-fluciclovine in MRMT-1 cells, a rat BCa cell line. We confirmed that ASCT2 and LAT1 were the primary AATs for ^14^C-fluciclovine transport in MRMT-1 cells, as well as prostate and brain cancer cell lines [8,9,10,11,12]. Second, we designed a bone metastasis model and injected MRMT-1 cells into the saphenous artery of the hind legs of rats. In this model, the mixed lesions of osteolytic and osteoblastic metastases, closely mimicking clinical findings [7], were formed in the tibia and/or femur.

Using this animal model, we performed triple-tracer autoradiography with ^14^C-fluciclovine/^3^H-FDG/^99m^Tc-HMDP and evaluated the accumulation of each tracer in identical bony lesions. Comparing the T/BG ratios of ^14^C-fluciclovine accumulation in rats that were fasted overnight with those in rats fed ad libitum, the ratios were higher in both osteolytic and osteoblastic lesions under the fasted condition than in the fed condition. It has been reported that the sum concentration of all plasma AAs decrease by 10% in rats fasted for one day, although the decreases do not involve drastic changes, unlike blood glucose (−35%) [13]. Among the AAs, proline (−48.9%), alanine (−21.8%), histidine (−18.2%), and glutamine (−13.1%), which are substrates of the ASC or L systems, demonstrated relatively high reduction rates in this experiment [13]. Thus, we expected that the concentration of these neutral AAs in rat plasma would be decreased by fasting before the injection of ^14^C-fluciclovine and, consequently, that the autoradiography images of tumor lesions would be improved by increased ^14^C-fluciclovine transport into the cancer cells. The T/BG ratios of ^14^C-fluciclovine were, in fact, statistically higher under the fasted condition compared with the fed condition. Thus, we believe that it is reasonable to prescribe fasting to PCa patients before fluciclovine-PET, not only to decrease plasma AA concentration, but to force the AA concentration toward favoring fluciclovine transport, thus achieving a more quantitative fluciclovine-PET scan.

There have been some reports describing the influence of meal or plasma AAs on AA-tracer accumulation in tumors. Lindholm et al. [4] performed an intra-individual clinical study to investigate whether the ingestion of a liquid meal influenced the accumulation of ^11^C-methionine, which is transported by LAT1 [10,11], in patients with head and neck cancer. In that study, initial PET imaging was performed after an overnight fast; the second PET imaging was then performed after ingesting the liquid meal containing L-methionine and branched-chain AAs 6 to 7 days after the first PET imaging. PET imaging was initiated 45 min after ingestion. The standard uptake values of ^11^C-methionine in the tumor after ingestion decreased to a statistically lower level than that in tumors after fasting, although the tracer entering rate (i.e., K_1_) into the tumor was not changed substantially [4]. Additionally, ^11^C-methionine uptake in gliomas and normal brain tissue, while the patient received an intravenous infusion of branched-chain AAs, decreased [14]. Similar results were observed in glioma patients injected with 3-^123^I-Iodo-L-α-methyltyrosine (^123^I-3-IMT), which is known to be transported by LAT1, with intravenous infusion of a mixture of naturally-occurring L-AAs during imaging [15]. The decreasing level of tracer accumulation in tumors observed in these three studies was believed to be caused by competitive inhibition between the exogenous AAs and tracers on LAT1. Thus, it is believed that ingesting food containing abundant AAs before, or intravenous infusion of AAs during, fluciclovine-PET imaging decreases the tumor accumulation of ^18^F-fluciclovine in PCa patients because ^18^F-fluciclovine is also transported by LAT1.

ASCT2 and LAT1 are obligatory AA exchangers with a 1:1 stoichiometry. If cancer cells are preloaded with AAs, which can trans-stimulate the influx of extracellular AAs (trans-stimulators), the velocity of AA transport through the AA exchangers is accelerated [8]. Thus, the preinjection of trans-stimulators into cancer patients would be expected to accelerate the uptake of PET tracers into cancer cells. Based on this hypothesis, Lahoutte et al. investigated the effect of intraperitoneal preinjection (30 min before tracer injection) of each naturally-occurring AA (arginine, aspartate, glutamate, phenylalanine, proline, tryptophan) on the uptake of ^123^I-3-IMT into the subcutaneous tumor of Rhabdomyosarcoma (R1M) tumor-bearing rats fasted for 4 h [16]. They found that the strongest effect was observed with the preinjection of tryptophan, which is a substrate of LAT1, although all AAs used in the study accelerated the uptake of ^123^I-3-IMT into the tumor. This increase of ^123^I-3-IMT transport into the subcutaneous tumor is explainable and based on trans-stimulation by preloaded tryptophan via LAT1. Accordingly, it is believed that ^18^F-fluciclovine transport also would be accelerated by the preinjection of trans-stimulators because this PET tracer is also recognized by ASCT2 and LAT1. On the other hand, Lahoutte’s findings are discrepant with findings involving ^11^C-methionine reported by Lindholm et al. [4]. The reason for the discrepancy may be the result of differences in experimental protocols between the studies: the injected substance (liquid meal vs. AA), the injection route (oral vs. intravenous), the species (rats vs. human), and fasting duration before tracer injection (4 h vs. non-fasting). Although fasting and preloading AAs appear to be conflicting treatments, there is a possibility that both may improve the visualization of PCa on fluciclovine-PET. Sophisticated, non-clinical studies are needed to determine the optimal imaging conditions for fluciclovine-PET, including variables such as fasting duration, type and dose of trans-stimulators, and timing of ^18^F-fluciclovine administration after fasting, or preinjection of trans-stimulators, because the metabolism of AAs in rats differs from that in humans.

We demonstrated that the T (tumor)/BG (muscle) ratios of ^14^C-fluciclovine in osteolytic and osteoblastic lesions in fasted rats were higher than in rats that were fed. We believe that the increased rate of ^14^C-fluciclovine uptake in cancer cells was greater than in muscular cells under experimental starvation. Our hypothesis for these findings is as follows: the most abundant and important neutral AA for mammalian cells is glutamine, which is transported by ASCT2 and sodium-dependent neutral amino acid transporter 2 (SNAT2) [17]. In muscle, SNAT2 is abundantly expressed compared with ASCT2 [18], and regulates intramuscular glutamine concentration [19,20,21]. A portion of intracellular glutamine is used for transport exchange with extracellular leucine via LAT1, and leucine regulates the production and degradation of protein in muscle as an activator for the mammalian target of rapamycin complex 1 (mTORC1), a key player in cellular functions such as protein and lipid synthesis, glycolysis, autophagy, etc. [22,23]. Decreases in intramuscular leucine represses mTORC1 signaling under fasting conditions, followed by the activation of the autophagic pathway, protein degradation, and the production of AAs [24]. The AAs are released from muscle and delivered to the liver and used for glycogenesis [25]. Thus, the efflux of AAs from muscular cells is facilitated under the starvation condition.

On the other hand, many types of cancers co-express ASCT2 and LAT1 on the cell surface [26], and these AATs function as key players for AA transport including glutamine and leucine in cancer cells [17]. In fact, many studies have reported that the co-expression of ASCT2 and LAT1 correlates with malignancy and prognosis in several types of cancer [27,28,29,30,31,32,33]. Although cancer cells can also actuate an autophagic process during starvation, the AAs produced by protein degradation in cancer cells are used for cancer-cell survival [34]. Instead, cancer facilitates the autophagic process in muscle tissue [35]. Thus, it is believed that cancer cells accelerate the influx of AAs rather than efflux, unlike muscle under fasting conditions. Because ASCT2 and LAT1 are the primary AATs mediating ^14^C-fluciclovine transport, we speculate that the increased rate of ^14^C-fluciclovine uptake in cancer cells is greater than in muscle cells under conditions of starvation. Therefore, the T/BG ratios of ^14^C-fluciclovine in the bone metastatic lesions in fasted rats were higher than in rats that were fed.

As mentioned, AA metabolism is a complex process, and different in humans and rats. The duration of fasting for fluciclovine-PET (4 to 6 h) scans in clinical settings and our animal experiment (17 to 18 h) is quite different. Thus, there is a possibility that the influence of fasting on ^18^F-fluciclovine accumulation in cancer tissue is reduced in clinical practice compared with the influence observed in our animal experiments. Additionally, the imaging time in the current study was 30 min, which falls within the plateau phase of ^18^F-fluciclovine uptake in orthotopic transplanted PCa of a previously described animal model [36]. On the other hand, imaging results at 30 min correspond to the late phase of a clinical fluciclovine-PET scan (typically 5 to 30 min); by that time, ^18^F-fluciclovine uptake decreases in prostate, lymph node, and bone lesions [37,38]. Consequently, uptake may have been underestimated in the current animal experiment. Further in vitro and in vivo studies are needed to extrapolate our findings to humans. Furthermore, intra-individual clinical studies are required to elucidate whether fasting and/or preloading trans-stimulators before fluciclovine-PET influences tumor accumulation of ^18^F-fluciclovine and improves fluciclovine-PET imaging in cancer patients.

## 4. Materials and Methods

### 4.1. Reagents

All reagents were purchased from Life Technologies (Carlsbad, CA, USA), Sigma-Aldrich (St. Louis, MO, USA), Nacalai Tesque (Kyoto, Japan), and Wako Pure Chemical Industries (Osaka, Japan), unless otherwise indicated. ^14^C-labeled fluciclovine (^14^C-fluciclovine) and ^3^H-labeled FDG (^3^H-FDG) were used instead of ^18^F-labeled tracers because their long half-lives make them more suitable for experiments than tracers labeled with ^18^F (t ½ = 110 min). ^14^C-Fluciclovine (specific activity, 2.08 GBq/mmol; radiochemical purity (RCP) >98%), which has the same chemical structure as ^18^F-fluciclovine, with the exception of the position of the radioisotope, was commercially synthesized by Nemoto Science (Tokyo, Japan), as described previously [8]. ^3^H-FDG (specific actvity 2.22 TBq/mmol; RCP, >99%) was purchased from American Radiolabeled Chemicals (St. Louis, MO, USA). The authors’ company, Nihon Medi-Physics (Tokyo, Japan), produced ^99m^Tc-HMDP (740 MBq/vial; RCP, >95%). All AAs used in this study were the L-form.

### 4.2. Cell Culture

A rat BCa cell line, MRMT-1, was procured from RIKEN BioResource Center (Tsukuba, Japan; obtained in January 2014) and maintained in RPMI 1640 medium supplemented with 10% fetal bovine serum (American Type Culture Collection, Rockville, MD, USA), 100 μg/mL streptomycin, and 100 U/mL penicillin.

### 4.3. Competitive Inhibition Assay

The contributions of Na^+^-dependent and -independent AATs to ^14^C-fluciclovine transport was estimated using competitive inhibition assays between ^14^C-fluciclovine and naturally-occurring or synthetic AAs, as described in a previous report [8]. Briefly, MRMT-1 cells were cultured in 24-well, flat-bottom tissue culture plates, and culture medium was replaced with sodium buffer (140 mmol/L NaCl, 5 mmol/L KCl, 5.6 mmol/L glucose, 0.9 mmol/L CaCl_2_, 1.0 mmol/L MgCl_2_ and 10 mmol/L HEPES (pH 7.3)) or choline buffer (NaCl of the sodium buffer was preplaced with 140 mmol/L choline chloride) containing 10 μmol/L ^14^C-fluciclovine in the presence or absence of 2.0 mmol/L inhibitor. Cells were placed in an incubator at 37 °C for 5 min, and radioactivity was measured using a liquid scintillation counter (Tri-Carb 2910TR, Perkin Elmer, Waltham, MA, USA). Protein concentrations of cell lysates were determined using the BCA Protein Assay Kit (Thermo Fisher Scientific, Waltham, MA, USA). Tracer uptake was calculated as pmol/mg protein and the control transport (absence of inhibitors) of ^14^C-fluciclovine in sodium/choline buffer was normalized to 100%. In this study, the following synthetic and naturally-occurring AAs were used as inhibitors: BCH, MeAIB, phenylalanine, proline, glutamine, serine, arginine, and glutamate; the specificity of each AA to AATs have been described in a previous report [8].

### 4.4. Animal Handling

All animal handling procedures and experimental protocols were conducted in accordance with Japanese laws as stipulated in the Act on Welfare and Management of Animals, and approved by the Institutional Animal Care and Use Committee of Nihon Medi-Physics (No. 142-019) on 24 October 2014. Male Sprague-Dawley rats (9–12 weeks old; CLEA Japan, Tokyo, Japan) were used for all experiments. Rats were housed in a 12 h light-dark cycle and were maintained on a standard laboratory diet, Labo MR Stock (Nosan Corporation, Kanagawa, Japan) containing approximately 9.2% water, 18.8% crude protein, 3.9% crude fat, 6.6% crude fiber, 6.9% crude ash, 54.7% of nitrogenous compounds, amino acids (1.09% arginine, 0.46% histidine, 0.67% isoleucine, 1.36%, leucine, 0.89%, lysine, 0.26% methionine, 0.84% phenylalanine, 0.64% threonine, 0.21% tryptophan, 0.79% valine, 0.28% cystein), and drinking water. All animals were anesthetized using 1% isoflurane (Pfizer Japan, Tokyo, Japan). In the surgical procedures, 0.5% meloxicam (Boehringer Ingelheim Vetmedica, Tokyo, Japan) was injected subcutaneously to relieve pain.

### 4.5. BCa Bone Metastatic Model

A BCa bone metastatic model, described previously [39], was used. Briefly, 100 mL of a cell suspension containing 2.5 × 10^4^ MRMT-1 cells in Hank’s Balanced Salt Solution without Ca^2+^ and Mg^2+^ was injected into the saphenous artery of the right hind legs. Development of osteolytic lesions was monitored using a microfocus X-ray imaging system (μFX-1000; FUJIFILM Corporation, Tokyo, Japan) or a preclinical imaging system (FX3000 CT, TriFoil Imaging, Chatsworth, CA, USA) and triple-tracer autoradiography was performed 12 ± 1 days after the cell injection. Osteolytic lesions were induced in the tibia and/or femur of the experimental rats.

### 4.6. Triple-Tracer Autoradiography

To compare the distribution of each tracer visually in identical lesions, triple-tracer autoradiography was performed using ^14^C-fluciclovine, ^3^H-FDG, and ^99m^Tc-HMDP in the BCa osteolytic model as described in the supplementary material. Briefly, rats with (*n* = 5) or without (*n* = 6) fasting overnight (17 h to 18 h) were injected (tail vein) with three tracers (^14^C-fluciclovine: 2.75 MBq/kg, ^3^H-FDG: 18.5 MBq/kg, ^99m^Tc-HMDP: 74 MBq/kg). ^14^C-Fluciclovine and ^3^H-FDG were allowed to remain in circulation for 30 min; ^99m^Tc-HMDP was allowed to remain for 2 h before euthanizing the animal. The tibiae and femora were removed and frozen in isopentane/dry ice; the frozen bone was then embedded in Super Cryoembedding Medium (SCEM) (Section-Lab, Hiroshima, Japan), followed by freezing again in isopentane/dry ice until the SCEM set. The frozen samples were sectioned using a CM3050S cryostat (Leica Microsystems, Tokyo, Japan) at −20 °C with an adhesive film (Cryofilm Type 2C(9), Section-Lab) using Kawamoto’s film method (5 μm slices and 10 μm slices for the histological and autoradiography specimens, respectively) [40]. The frozen samples were provided for triple-tracer autoradiography and each ^14^C-, ^3^H-, and ^99m^Tc-image was created as described in the supplementary materials. The regions-of-interest (ROIs) were manually drawn around each lesion while referring to the histological images from H&E and TB staining. In the ^14^C-fluciclovine and ^3^H-FDG images, tumor lesions with bone absorption were defined as OL (typical osteolytic lesions) and the lesions corresponding to ^99m^Tc-HMDP accumulation were defined as peri-tumor bone formation (pTBF) in the osteoblastic lesions (typical osteoblastic lesions). Furthermore, three ROIs of random size were manually positioned on the normal regions of muscle surrounding tibiae and/or femora and the average ROI count from the three ROIs was calculated as the background radioactivity. The target_mean_ (metastatic lesion)-to-background_mean_ (muscle) (T/BG) ratios were then calculated and lesion-based analyses were performed.

### 4.7. Histological Analysis

The following procedures for each histological stain were performed on 5 μm serial sections using general methods: H&E and TB staining for histological changes at the lesion sites; ALP for osteoblast and fibroblast activity; and TRAP staining for osteoclast-activity. Anti-ASCT2 (J-25) polyclonal antibody (1:40; Santa Cruz Biotechnology, Dallas, TX, USA) and anti-LAT1 (H-75) polyclonal antibody (1:200; Santa Cruz Biotechnology) for the amino acid transporters were used in the immunohistochemical assessments with EnVision+ System HR-labeled polymer anti-rabbit (Dako Japan, Tokyo, Japan) and Liquid DAB+ Substrate Chromogen System (Dako Japan). A BZ-9000 HS all-in-one fluorescence microscope (Keyence Corporation, Osaka, Japan) was used for pathological examinations.

### 4.8. Statistical Analysis

Data are presented as mean ± the standard deviation. To create beeswarm plots with box plots, R version 3.3.2 (R Foundation, Vienna, Austria) for Windows (Microsoft Corporation, Redmond, WA, USA) was used. In these plots, each dot represents a T/BG ratio in each lesion and the center lines in the boxes represent the medians; box limits indicate the 25th and 75th percentiles; whiskers extend 1.5 times the interquartile range from the 25th and 75th percentiles. All statistical analyses were performed using SAS version 9.4 (SAS Institute, Cary, NC, USA). The two groups were compared using Wilcoxon rank-sum tests for non-normal distribution datasets, or the F-test, followed by two-tailed unpaired Student’s *t*-test or Welch’s *t*-test for datasets with normal distribution. In all analyses, *p* < 0.05 was considered to be statistically significant.

## 5. Conclusions

Our findings suggest that fasting influences the uptake of ^18^F-fluciclovine in osteolytic and osteoblastic bone metastasis lesions, and can facilitate clearer visualization of lesions in fluciclovine-PET imaging. However, because AA metabolism is a complex process, further animal and clinical studies are required to confirm our findings.

## Figures and Tables

**Table 1:** Summary of the distributions of T/BG ratios of ^3^H-FDG and ^14^C-fluciclovine in the osteolytic lesions and pTBF in osteoblastic lesions of breast cancer bone metastasis model rat with and without overnight fasting.

Lesion	Tracer	Diet	Min.	Max.	Median	1st Qu.	3rd Qu.	Mean ± S.D.
OL	^3^H-FDG	Fasting	2.9	13.2	6.2	5.5	7.0	6.9 ± 2.5
		Feeding	1.6	10.7	5.0	3.7	6.1	5.1 ± 2.0
	^14^C-fluciclovine	Fasting	3.1	6.8	4.4	3.9	5.1	4.6 ± 0.8
		Feeding	1.4	5.9	2.7	2.4	3.1	2.8 ± 0.6
pTBF	^3^H-FDG	Fasting	1.6	12.8	5.6	3.7	10.5	6.6 ± 3.8
		Feeding	0.6	8.2	2.8	1.7	4.3	3.3 ± 2.2
	^14^C-fluciclovine	Fasting	2.6	7.6	4.1	3.8	4.9	4.5 ± 1.4
		Feeding	1.0	3.9	2.5	1.8	2.9	2.4 ± 0.7

1st Qu.: first quartile, 3rd Qu.: third quartile, ^3^H-FDG: 2-deoxy-2-^18^F-fluoro-d-glucose, Max.: maximum value of T/BG ratios, Min.: minimum value of T/BG ratios, pTBF: peri tumor bone formation, OL: osteolytic, S.D.: standard deviation, T/BG: target_mean_ (metastatic lesion)-to-background_mean_ (muscle).

## Supplementary Materials

Supplementary materials can be found at www.mdpi.com/1422-0067/18/5/934/s1.

## Abbreviations

AA	amino acid
AAT	amino acid transporter
ALP	alkaline phosphatase
ASC	alanine-serine-cysteine
ASCT2	alanine-serine-cysteine transporter 2
BCa	breast cancer
BM	bone marrow
^11^C	carbon-11
^14^C	carbon-14
CB	cortical bone
^18^F	fluorine-18
FDG	2-deoxy-2-fluoro-d-glucose
^3^H	tritium-3
H&E	hematoxylin-eosin
HMDP	hydroxymethylene diphosphonate
LAT1	L-type amino acid transporter 1
mTORC1	mammalian target of rapamycin complex 1
^99m^Tc	technetium-99m
Ob	osteoblasts
Oc	osteoclasts
Os	osteoids
PET	positron emission tomography
PCa	prostate cancer
pTBF	peri-tumor bone formation
RCP	radiochemical purity
ROI	region-of-interest
SCEM	Super Cryoembedding Medium
SNAT2	sodium-dependent neutral amino acid transporter 2
TB	toluidine blue
T/BG	target_mean_ (metastatic leion)-to-background_mean_ (muscle)
TRAP	tartrate-resistant acid phosphatase
