# Supplementary material for: Fasting Enhances the Contrast of Bone Metastatic Lesions in 18F-Fluciclovine-PET: Preclinical Study Using a Rat Model of Mixed Osteolytic/Osteoblastic Bone Metastases
Source: Int J Mol Sci. 2017 Apr 29;18(5):934. doi: 10.3390/ijms18050934 (PMC5454847; doi:10.3390/ijms18050934)
Supplement: Supplementary file 1 [file ijms-18-00934-s001.pdf]

Supplementary Materials: Fasting Enhances the Contrast of Bone Metastatic Lesions in 18F-Fluciclovine-PET: Preclinical Study Using a Rat Model of Mixed Osteolytic/Osteoblastic Bone Metastases

Shuntaro Oka, Masaru Kanagawa, Yoshihiro Doi, David M. Schuster, Mark M. Goodman and Hirokatsu Yoshimura

Materials and Methods

Triple-tracer autoradiography

Triple-tracer autoradiography was performed using *trans*-1-amino-3-fluoro [1-^{14}C] cyclobutanecarboxylic acid (^{14}C-fluciclovine), [5,6-^{3}H]-2-Fluoro-2-deoxy-D-glucose (^{3}H-FDG), and ^{99m}Tc-hydroxymethylene diphosphonate (^{99m}Tc-HMDP) in a breast cancer bone metastatic rat model to visually compare the distribution of each tracer in identical lesions. After administering the tracers (see article text), the animals were euthanized under anesthesia by drawing blood from the abdominal aorta. The tibiae and femora were removed and subsequently frozen in isopentane/dry ice for 10 s. They were embedded in Super Cryoembedding Medium (SCEM) (Section-Lab, Hiroshima, Japan) and frozen again in isopentane/dry ice until the SCEM set. The frozen samples were placed in the chamber of a CM3050S cryostat (Leica Microsystems, Tokyo, Japan) at -20°C for at least 30 min and sectioned using the cryostat at −20 °C with an adhesive film (Cryofilm Type 2C(9), Section-Lab) using Kawamoto's film method [1]. Fifteen serial sections (12 sections of 5 μm slices, and 3 sections of 10 μm slices for the histological and autoradiography specimens, respectively) were prepared. Each section was mounted on a glass slide. To obtain images generated by the ^{99m}Tc-isotope, SR imaging plates (IPs) (FUJIFILM Corporation, Tokyo, Japan) were exposed for 1 h to dried 10 μm slices wrapped in a 12 μm thick polyester film (Lumirror; S10#12; TORAY Industries, Tokyo, Japan), which absorbs low-energy ^{3}H isotopes. Under these conditions, ^{14}C caused no blackening of the SR-IP, even after a 1 h exposure. This factor, thus, excluded cross-contamination by ^{14}C in the ^{99m}Tc autoradiographs. The next two frozen sections adjacent to the ^{99m}Tc-autoradiographed section were stored at −20°C for 5 days to allow complete ^{99m}Tc decay. Following this procedure, TR-IPs (FUJIFILM Corporation) were exposed to dried sections with and without the 12 μm thick polyester film for 7 days to obtain ^{14}C images and ^{3}H + ^{14}C mixed images, respectively [2]. The IPs were developed using a FLA-7000 imaging analyzer (GE Healthcare, Little Chalfont, UK). The ^{3}H images were finally generated by subtracting the ^{14}C images from the ^{14}C + ^{3}H images using ImageJ software (version 1.48; National Institutes of Health, Bethesda, MD, USA). All images were processed using ImageJ software. ^{14}C, ^{3}H, and ^{99m}Tc images obtained from a serial section were stacked in a single window using the "Stack" function of the ImageJ software. For image registration of the three images, each was positioned precisely by using several internal soft/hard tissue landmarks with characteristic anatomical information (except for lesions), such as growth plate, a portion of cortical bone of tibiae/femora (ex. distal end), patellae, cartilaginous tissue, and the region-of-interest (ROI) analysis was conducted. The ROIs were manually drawn around each lesion while referring to the histological images from hematoxylin-eosin (H&E) and toluidine blue (TB) staining. In each model, the lesions corresponding to ^{99m}Tc-HMDP accumulation, except for physiological accumulation (e.g., growth plates, the tendon at the tibia) were defined as new bone formation. Furthermore, three square, circular, or polygonal ROIs of random sizes were manually positioned on the normal regions of muscle surrounding tibiae and/or femora (ex. quadriceps femoris muscle, gastrocnemius muscle) and the average ROI count from the three ROIs was calculated as the background radioactivity.

Results

Histological analysis

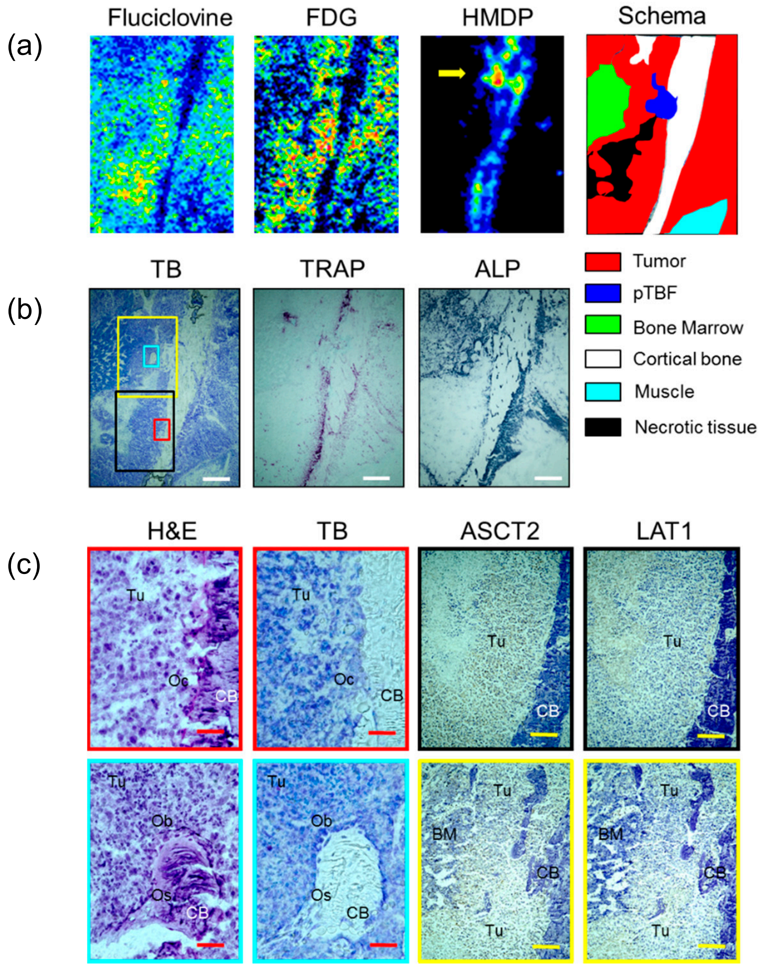

Figure S1. Comparison of tracer accumulations of ^{14}C-fluciclovine, 2-deoxy-2-^{18}F-fluoro-D-glucose (^{3}H-FDG) and ^{99m}Tc-hydroxymethylene diphosphonate (^{99m}Tc-HMDP), and the histological characteristics of typical osteolytic and osteoblastic lesions in a representative breast cancer bone metastasis model rat that was fasted. (**a**) The enlarged autoradiograms and schema and (**b**) the histological images (toluidine blue (TB), tartrate-resistant acid phosphatase (TRAP), alkaline phosphatase (ALP)) correspond to the green frame on the schema in Figure 2a are represented. The lesions corresponding to ^{99m}Tc-HMDP-positive were considered peri-tumor bone formation (pTBF) in osteoblastic lesions (yellow arrows). (**c**) The high-power microscopic fields (hematoxylin and eosin (H&E), TB, alanine-serine-cysteine transporter 2 (ASCT2), L-type amino acid transporter 1 (LAT1)) correspond to the black, red, yellow, and cyan frames on the TB image in Figure S1b are shown. The red, yellow, and white scale bars on each panel correspond to 50 μm, 200 μm, and 500 μm, respectively. BM: bone marrow, CB: cortical bone, Ob: osteoblasts, Oc: osteoclasts, Os: osteoids, Tu: tumor.

References

1. Kawamoto, T. Use of a new adhesive film for the preparation of multi-purpose fresh-frozen sections from hard tissues, whole-animals, insects and plants. *Arch. Histol. Cytol.* **2003**, *66*, 123–143.
2. Obata, T.; Iwamoto, K.; Shiraiwa, Y.; Nakajima, E.; Kawai, K.; Shindo, H. Instruments for radiation measurement in biosciences. Series 3. Radioluminography. 17. Analysis of double-labelled samples by the imaging plate (IP). *Radioisotopes* **2000**, *49*, 623–636.
